# Influence of doxorubicin on model cell membrane properties: insights from *in vitro* and *in silico* studies

**DOI:** 10.1038/s41598-017-06445-z

**Published:** 2017-07-24

**Authors:** Ana Catarina Alves, Aniket Magarkar, Miguel Horta, Jose L. F. C. Lima, Alex Bunker, Cláudia Nunes, Salette Reis

**Affiliations:** 1 UCIBIO, REQUIMTE, Departamento de Ciências Químicas, Faculdade de Farmácia, Universidade do Porto, Porto, Portugal; 2 Institute of Organic Chemistry and Biochemistry, Academy of Sciences of the Czech Republic, Flemingovo nám. 2, 16610 Prague 6, Czech Republic; 3 Faculty of Pharmacy, University of Helsinki, Viikinkaari 5E, Helsinki, 00014 Finland

## Abstract

Despite doxorubicin being commonly used in chemotherapy there still remain significant holes in our knowledge regarding its delivery efficacy and an observed resistance mechanism that is postulated to involve the cell membrane. One possible mechanism is the efflux by protein P-gp, which is found predominantly in cholesterol enriched domains. Thereby, a hypothesis for the vulnerability of doxorubicin to efflux through P-gp is its enhanced affinity for the ordered cholesterol rich regions of the plasma membrane. Thus, we have studied doxorubicin’s interaction with model membranes in a cholesterol rich, ordered environment and in liquid-disordered cholesterol poor environment. We have combined three separate experimental protocols: UV-Vis spectrophotometry, fluorescence quenching and steady-state anisotropy and computational molecular dynamics modeling. Our results show that the presence of cholesterol induces a change in membrane structure and doesn’t impair doxorubicin’s membrane partitioning, but reduces drug’s influence on membrane fluidity without directly interacting with it. It is thus possible that the resistance mechanism that lowers the efficacy of doxorubicin, results from an increased density in membrane regions where the efflux proteins are present. This work represents a successful approach, combining experimental and computational studies of membrane based systems to unveil the behavior of drugs and candidate drug molecules.

## Introduction

**Figure 1:**
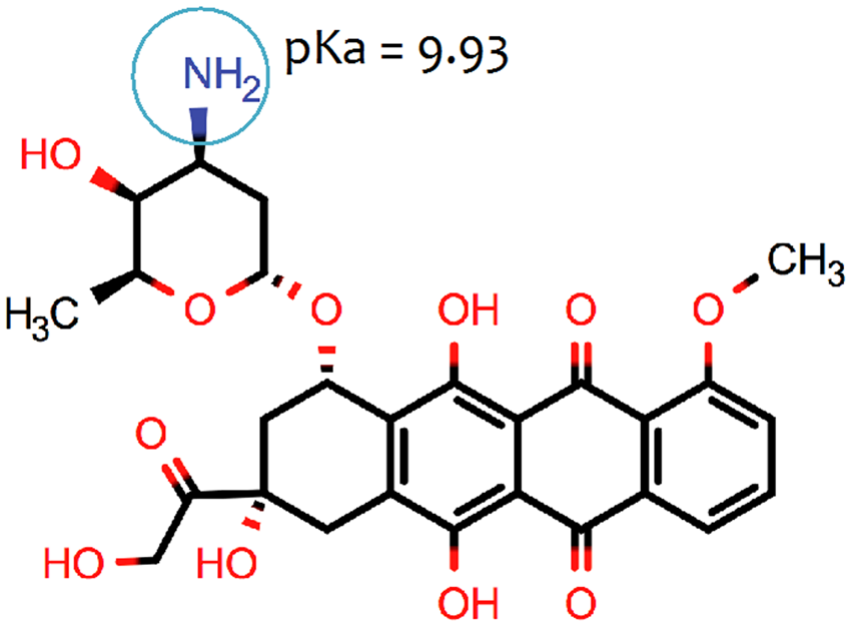
Molecular structure and pKa value of doxorubicin.

Doxorubicin (as shown in Fig. 1) is one of the most widely prescribed anticancer drugs^1^. The most prevalent opinion is that the anticancer activity of the drug is mainly due to direct interaction with nucleic acids, leading to DNA damage and inhibition of DNA synthesis. This is, however, still the subject of considerable debate^1^; there is also strong evidence suggesting that interaction with cell membranes plays a role in its activity^2^. Clearly, even if the dominant mechanism is the interaction with the nucleic acids, doxorubicin must pass through a variety of other organelles to reach the DNA^3^. First, the drug must pass the selective barrier to entry into the cell that is the cell membrane and, finally, the nuclear membrane. Thereby, interaction with lipid membranes is an unavoidable step in doxorubicin activity, whatever the exact mechanism of action proves to be.

Drug-membrane interactions produce alterations in the physical properties of the cell membrane, as a result of the behavior of the drug within the membrane. How the drug orients in the membrane, which components of the lipid membrane the drug interacts with and what interactions within the membrane the drug disrupts, will all alter the physical properties of the membrane^4^. This can, in turn, lead to changes in cell signaling and the function of membrane proteins, e.g. transporters and ion channels^5^. It was, for example, found that Daunorubicin (a doxorubicin analog) causes alteration of both G-proteins and protein kinase C-associated signaling pathways, through destabilizing the non-lamellar membrane structures that are involved in their location and activity^6, 7^. As these proteins participate in fundamental functions of the cell, including proliferation and differentiation, their modulation could be responsible for the antitumor activity of some drugs^6^.

Within the membrane, the lipids are heterogeneously distributed into dynamic domains; domains enriched in cholesterol and sphingolipids are thought to form a highly ordered, liquid-ordered phase (l_o_), *rafts* that metaphorically float in a liquid-disordered phase (l_d_) matrix^8^. The function of several membrane proteins is highly dependent on their association with these domains^9, 10^. Among the proteins thought to be associated with lipid rafts^11^ is P-glycoprotein (P-gp)^12^ that effluxes a variety of hydrophobic, neutral, and positively charged drugs from the cell. P-gp is a component of the normal cellular defense system against xenobiotics^13^. This protein has been shown to be highly sensitive to membrane fluidity; thus, any changes in membrane fluidity will alter the ability of P-gp to efflux drug molecules^11^.

Doxorubicin is among the substrates effluxed by P-gp; thus, in this case, this protein plays a key role in drug resistance^14^. What is not so well understood, however, is: 1) how doxorubicin partitions between the l_o_ rafts and the l_d_ matrix and 2) how doxorubicin alters the physical properties of the lipid rafts. This has considerable relevance regarding the behavior of doxorubicin as a drug. In fact, the manner in which doxorubicin partitions between the two phases of the plasma membrane will determine its susceptibility to P-gp: the greater the extent to which doxorubicin partitions to the P-gp rich rafts the greater its susceptibility to efflux. Additionally, the effect of the presence of doxorubicin on the structure of the l_o_ raft can not only effect P-gp function, but also result in the modulation of the physicochemical properties of the membrane itself. This could, in turn, initiate different processes implicated in the anticancer cytotoxic effects of the drug. Several anticancer drugs, in fact, induce modifications in the content of lipid rafts, leading to growth inhibition and activation of tumor cells apoptosis^9^.

In light of all of this, we set out to apply a combined protocol of *in vitro* experimental analysis techniques, including UV-Vis spectrophotometry, fluorescence quenching and steady state anisotropy, both using membrane bound fluorescent probes and *in silico* computational modeling, to study the interaction of doxorubicin with the plasma membrane. Properties studied include: 1) effect of doxorubicin on membrane fluidity, 2) distribution of doxorubicin between the l_o_ and l_d_ phases and, 3) through computational molecular dynamics modeling, the microscopic details of the membrane-doxorubicin interactions. We show that cholesterol, present in the l_o_ rafts but not so prevalent in the l_d_ matrix, alters the behavior of doxorubicin in the membrane without directly interacting with it. This interaction occurs in a fashion that promotes increased location of doxorubicin to the l_o_ rafts, possibly increasing uptake by the P-gp efflux protein. We propose that this could possibly be a mechanism that contributes to the doxorubicin chemoresistance problem.

## Results and Discussion

### Doxorubicin Partition Coefficients

The partitioning of drugs into biological membranes plays a significant role in their uptake, transport, bioavailability and distribution^15^. In fact, doxorubicin needs to cross the cell membrane to reach its intracellular targets, for example DNA, and the interaction with lipids can strongly influence the pharmacokinetic and pharmacodynamic properties^15^ of the drug. The partition coefficient (Kp), is an important property to determine, as it provides information concerning the distribution of drugs between aqueous and lipid phases, of direct relevance to the pharmacological activity of the drug. The doxorubicin partition coefficient in several model membranes was determined through derivative UV-Vis spectrophotometry. This technique, based on the fact that the spectral characteristics (λ_max_) of the drug change when it permeates from the aqueous to the lipid medium, allows for the quantification of drug distribution between each phase. Furthermore, it also provides a better resolution of the overlapped bands and eliminates the lipid light scattering interference through the use of the derivative method.

**Figure 2:**
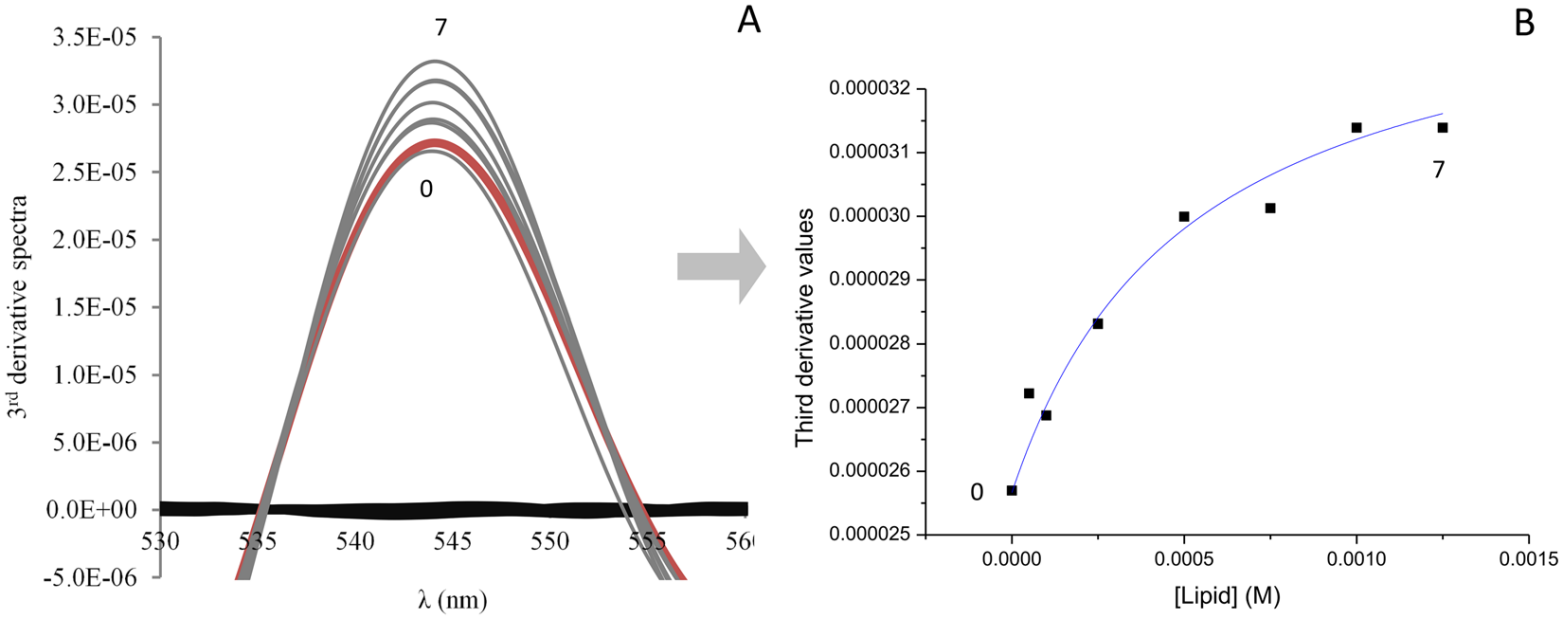
Third-derivative absorption spectra (**A**) of doxorubicin (40 μM) (red line, 0) alone, incubated in DMPC:SM model membrane at 37 °C with increasing lipid concentration (7 represents the maximum lipid concentration) and the model membrane without drug (black lines). (**B**) represents the best fitting curve to experimental third-derivative spectrophotometric data (DT vs. [L]) using a nonlinear regression method at a wavelength of 544 nm.

Figure 2(A) shows, as an example, the third-derivative absorption spectra of doxorubicin with different concentrations of liposomes composed of DMPC:SM [8:2] at pH 7.4 and at 37 °C. The use of the third-derivative allowed the elimination of the residual background signal caused by scattering from the liposomes. The best fit of equation 1 to the third-derivative data was achieved at 544 nm and is shown in Fig. 2(B).

The experimental values obtained for the doxorubicin partition coefficient with DMPC:SM and DMPC:SM:Chol (expressed as Kp and logD) are depicted in Table 1.

**Table 1:** Partition Coefficients (expressed as Kp and logD) of doxorubicin in DMPC:SM [8:2] and DMPC:SM:Chol [7:1.5:1.5] at pH 7.4 and 37 °C^a^.

Model	Kp	Experimental logD
DMPC:SM	8195 ± 406	3.91 ± 0.02
DMPC:SM:Chol	8707 ± 523	3.94 ± 0.03

^a^All values represent the mean ± standard deviation (n = 3).

^*^p < 0.05 statistically different from the other model.

Traditionally, the partition coefficient is assessed in octanol/water systems. The octanol/water logD of doxorubicin at pH 7.4, obtained using Marvin sketch calculator software (ChemAxon), was 0.02. Thereby, a lack of correlation between the doxorubicin partitioning coefficients obtained in liposome/buffer and octanol/water systems can be observed. Such difference is due to the fact that the biphasic octanol/water system can only account for hydrophobic interactions. Liposomes, on the other hand, provide an anisotropic environment that can better mimic membranes, which are composed by amphiphilic phospholipids and can, therefore, establish electrostatic and hydrophobic interactions. In addition, the pK_a_ value (9.93), calculated using Marvin sketch calculator software, predict that nearly 80% of doxorubicin molecules are in the cationic form at pH 7.4, providing evidence that not only hydrophobic intermolecular forces drive the doxorubicin’s partitioning. In fact, the octanol/water partition coefficient value is much smaller than that calculated by the liposome/buffer system, since it only reflects the hydrophobic interactions of a small number of molecules that are in the neutral form at pH 7.4. Hence, the use of a liposomes/buffer system allows one to obtain more realistic information regarding doxorubicin lipophilicity and, therefore, it’s *in vivo* membrane partitioning.

Furthermore, the gathered experimental results reveal that doxorubicin exhibits a similar membrane partitioning with both DMPC:SM and DMPC:SM:Chol liposomes. This suggests that the positively charged doxorubicin interacts with the phospholipid polar head groups of both models and that the presence of Chol does not impair such partitioning.

### Drug location studies using different fluorescent probes

The location of doxorubicin in the membrane was tracked through fluorescence quenching of membrane bound probes (TMA-DPH and DPH), since this technique provides a measure of the accessibility of the drug to each probe^16^. Such knowledge is only possible to obtain due to the fact that the fluorophore position in the membrane is well defined and documented. Therefore, while DPH is deeply incorporated in the hydrophobic regions of the lipid bilayer^17^, TMA-DPH is reported to be anchored in the polar head groups region of phospholipids due to its charged group^18^. According to this, in the current study the steady-state fluorescence intensities and lifetimes were measured in liposomes labeled with TMA-DPH and DPH probes. The theory and equations behind this technique are presented in the supporting information.

**Figure 3:**
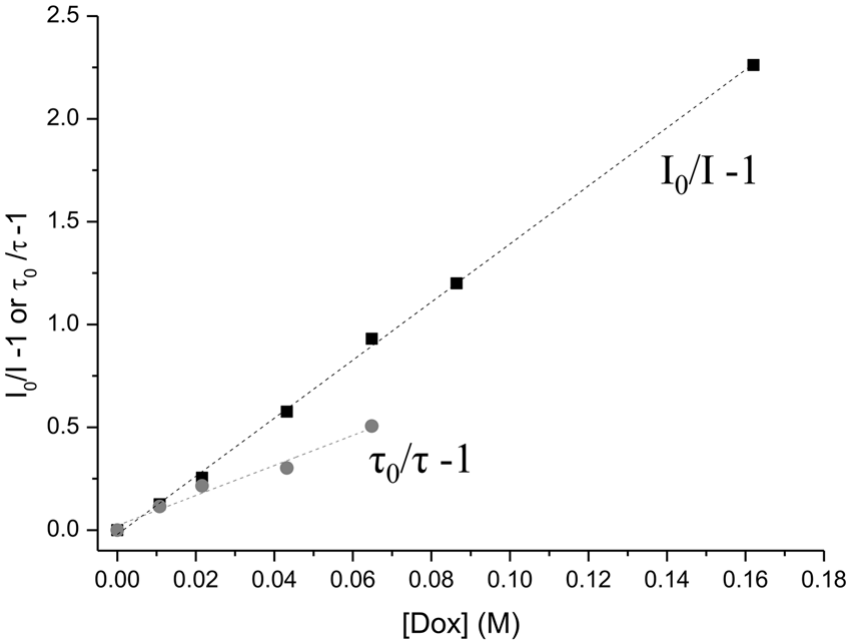
Stern–Volmer plots of the probe DPH in DMPC:SM [8:2] model at pH 7.4 and 37 °C with increasing doxorubicin concentrations: square symbols (◾) represent the Stern–Volmer plot obtained by steady-state fluorescence measurements (I_0_/I−1) and circle symbols () represent the Stern–Volmer plot obtained by lifetime fluorescence measurements (τ_0_/τ−1).

In Fig. 3, the Stern-Volmer plots of I_0_/I−1 and τ_0_/τ−1 as a function of doxorubicin membrane concentration are shown, for the DMPC:SM [8:2] model.

According to the description of the quenching behavior (see supporting information), the quenching parameter values, namely the dynamic constant (K_D_), the static constant (K_S_) and the Stern–Volmer constant (K_SV_), obtained from this technique are depicted in Table 2.

**Table 2:** Fluorescence quenching parameters (K_D_, dynamic constant; K_S_, static constant; K_SV_, Stern–Volmer constant) determined from the quenching of DPH and TMA-DPH by doxorubicin in the different mimetic models at pH 7.4 and 37 °C^a^.

Model	Probe	K_D_ (M^−1^)	K_S_ (M^−1^)	K_SV_ (M^−1^)
DMPC:SM	DPH	7.4* ± 0.2	6.4 ± 0.3	13.8* ± 0.4
	TMA-DPH	6* ± 1	6.0 ± 0.3	11* ± 1
DMPC:SM:Chol	DPH	3.2* ± 0.4	3.2* ± 0.2	6.4 ± 0.2
	TMA-DPH	2.5* ± 0.2	4.2* ± 0.2	6.6 ± 0.1

^a^All values represent the mean ± standard deviation (n = 3).

*p < 0.05 statistically different from the other probe, for each fluorescence quenching parameter.

From the analysis of Table 2, it is possible to conclude that the quenching process, in all mimetic model systems studied and probes used, results from both dynamic and static interactions. This behavior indicates that doxorubicin must diffuse into the fluorophore during the lifetime of the excited state; however, it can also form a non-fluorescent complex with the probe. According to the results, doxorubicin was able to quench both probes, DPH and TMA-DPH. Nevertheless, the K_SV_ values demonstrate that the decrease of the DPH fluorescence was more pronounced than for TMA-DPH in the membrane model composed of DMPC:SM [8:2]. This suggests that doxorubicin interacts with the lipid head groups of the phospholipids, but the drug’s dihydroanthraquinone residue may also interact with the lipid fatty acid chains through hydrophobic interactions. For the bilayer comprised by DMPC:SM:Chol [7:1.5:1.5] similar values of K_SV_ were obtained for both fluorescent probes, which means that doxorubicin has an identical accessibility to DPH and TMA-DPH in the model membranes with cholesterol present.

### Effect of Doxorubicin in membrane fluidity

Since doxorubicin interacts with membranes in order to exert its therapeutic action, it is important to understand how the compound can influence the physicochemical state of the phospholipid bilayer in order to explain its pharmacological action. Furthermore, alterations of membrane fluidity can severely affect the cell’s functional properties that might be correlated with the drug’s mechanism of action. In this context, membrane fluidity studies were performed by steady-state anisotropy^19^ using fluorescent probes (DPH and TMA-DPH). The ability of doxorubicin to disturb the membrane structure in different regions can be assessed since these two probes report the microfluidity of those sites^20^. Modifications in membrane fluidity can be detected by changes in anisotropy, which reflects perturbations in the probe’s rotational movement caused by the changes in the stiffness of its surrounding matrix^21^.

**Figure 4:**
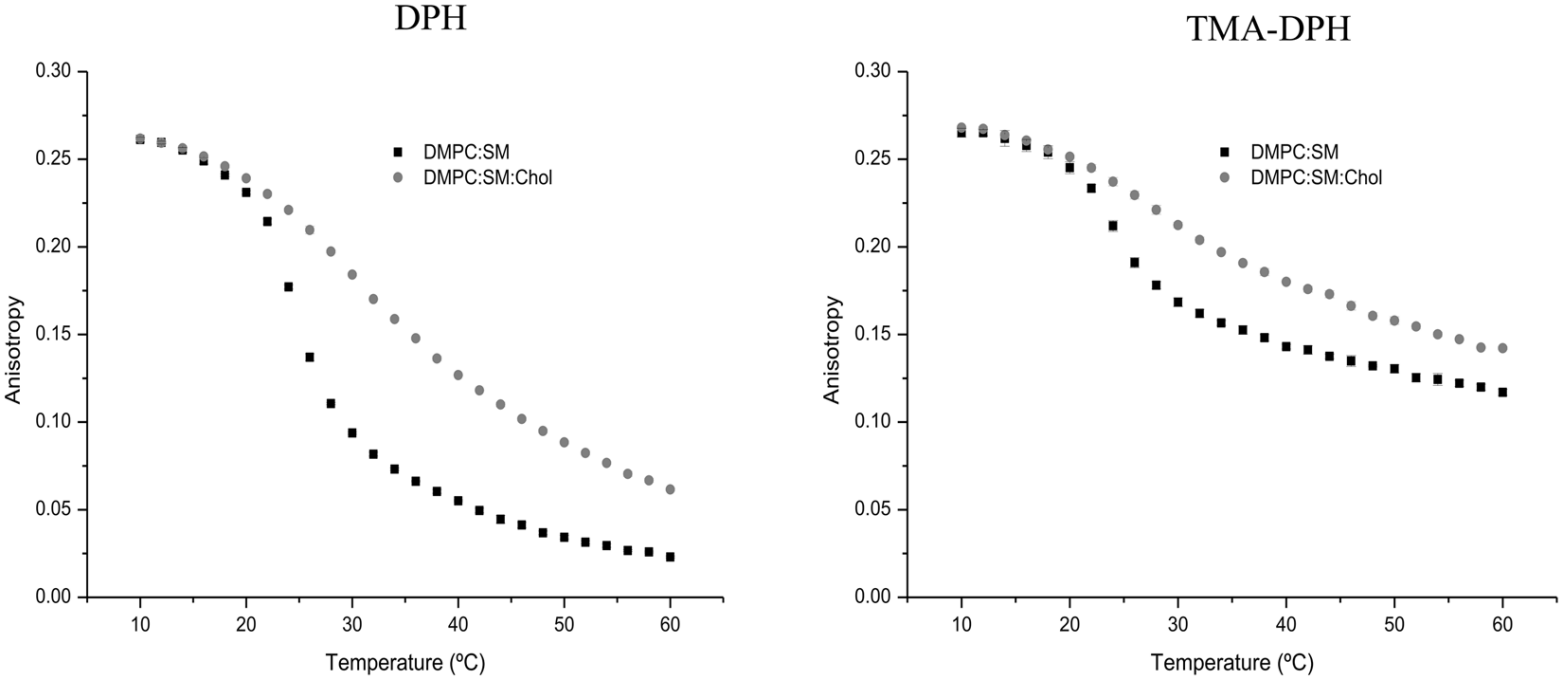
Steady-state anisotropy of DPH and TMA-DPH as a function of temperature in DMPC:SM [8:2] and DMPC:SM:Chol [7:1.5:1.5] membrane models. Results present the mean of at least three independent assays.

The effect of temperature on the DPH and TMA-DPH fluorescence anisotropy of the different model membranes without doxorubicin is presented in Fig. 4. The results demonstrate that the anisotropy values in both model membranes decrease with increasing temperature to a greater extent in the acyl chain region (given by the probe DPH) than in the phospholipid head region (given by the TMA-DPH probe). In fact, it is well documented that the membrane is characterized by a fluidity gradient from the aqueous interface to the bilayer interior, where the acyl chain end shows increased disorder. Also, it is known that the presence of SM and Chol in membranes promotes the formation and maintenance of specific domains in the liquid-ordered phase, more condensed and organized^22^, which results in higher anisotropy values.

**Figure 5:**
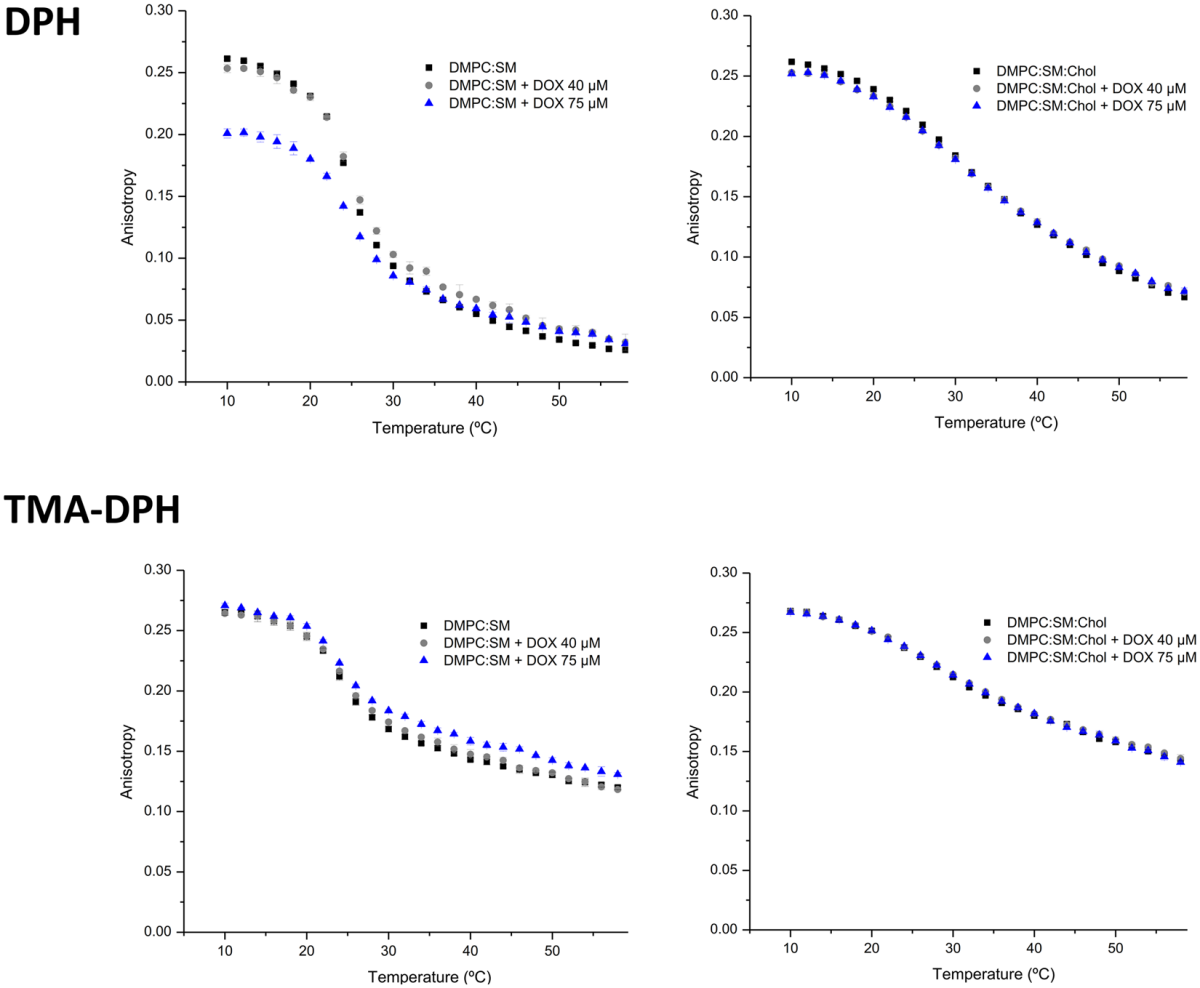
Steady-state anisotropy of DPH and TMA-DPH as a function of temperature in each mimetic model, in the absence (◾), and in the presence of doxorubicin 40 µM () and 75 µM (). Results present the mean of at least three independent assays.

Figure 5 shows the influence of doxorubicin on the DPH and TMA-DPH fluorescence anisotropy as a function of temperature for each model system studied. Regarding the model membrane composed of DMPC:SM two distinct doxorubicin behaviors can be observed in the different regions of the membrane. Namely, doxorubicin increased the membrane fluidity of the phospholipid’s acyl chains, in a more ordered phase of the bilayer, and decreased the membrane microviscosity, in a more fluid phase of the bilayer. This represents a similar behavior as Chol possesses when in incorporated in the membrane, helping to prevent the membrane from becoming either too fluid or too rigid, due to the ability of cholesterol to cause disordering of gel-phase lipids while still maintaining a high degree of orientational ordering. At the same time, DOX decreased the membrane microviscosity in the phospholipid’s head groups. These findings are, once again, consistent with the previous results of partition and location, where it has been observed that the anticancer drug establishes electrostatic interactions and hydrogen bonds with the negative phosphate group and hydrophobic interactions with the hydrocarbon chains of the phospholipids.

On the other hand, it can be observed that doxorubicin did not produced any alterations in the membrane fluidity of DMPC:SM:Chol model along the bilayer. This fact can be correlated with the disposition of the molecules within the phospholipid membrane. Doxorubicin is a molecule that possesses an aromatic ring wherein its planar structure bears resemblance to the structure of cholesterol. According to the location studies, doxorubicin has an identical accessibility to both DPH and TMA-DPH probes. Considering that the presence of Chol reduces the membrane fluidity, it is possible that doxorubicin does not cause significant alteration in the overall fluidity in both superficial and deeper regions of phospholipids. Furthermore, due to interactions between NH_3_^+^ of doxorubicin and the phosphate group in the polar head of the phospholipids, the further access of doxorubicin into the membrane might be impaired.

The presence of SM together with Chol form within the membrane, liquid-ordered domains that are characterized by a low density and rigid environment, as shown through anisotropy measurements. It has been demonstrated by several structure–activity studies that hydrophobicity and the presence of planar aromatic regions, as in the case of doxorubicin, favor the drug-P-gp interaction^23^. Furthermore, it was also reported that increase of membrane fluidity can lead to an inhibition of P-gp function^24^. Since our results demonstrate that doxorubicin did not cause any changes in the fluidity of the DMPC:SM:Chol model membrane and that the drug’s binding sites on the P-gp are via the lipid membrane^25^, it is possible to correlate the gathered outcomes with the resistance problem associated with doxorubicin administration.

### Molecular Dynamics simulation results: Interaction of doxorubicin with membrane bilayers

**Figure 6:**
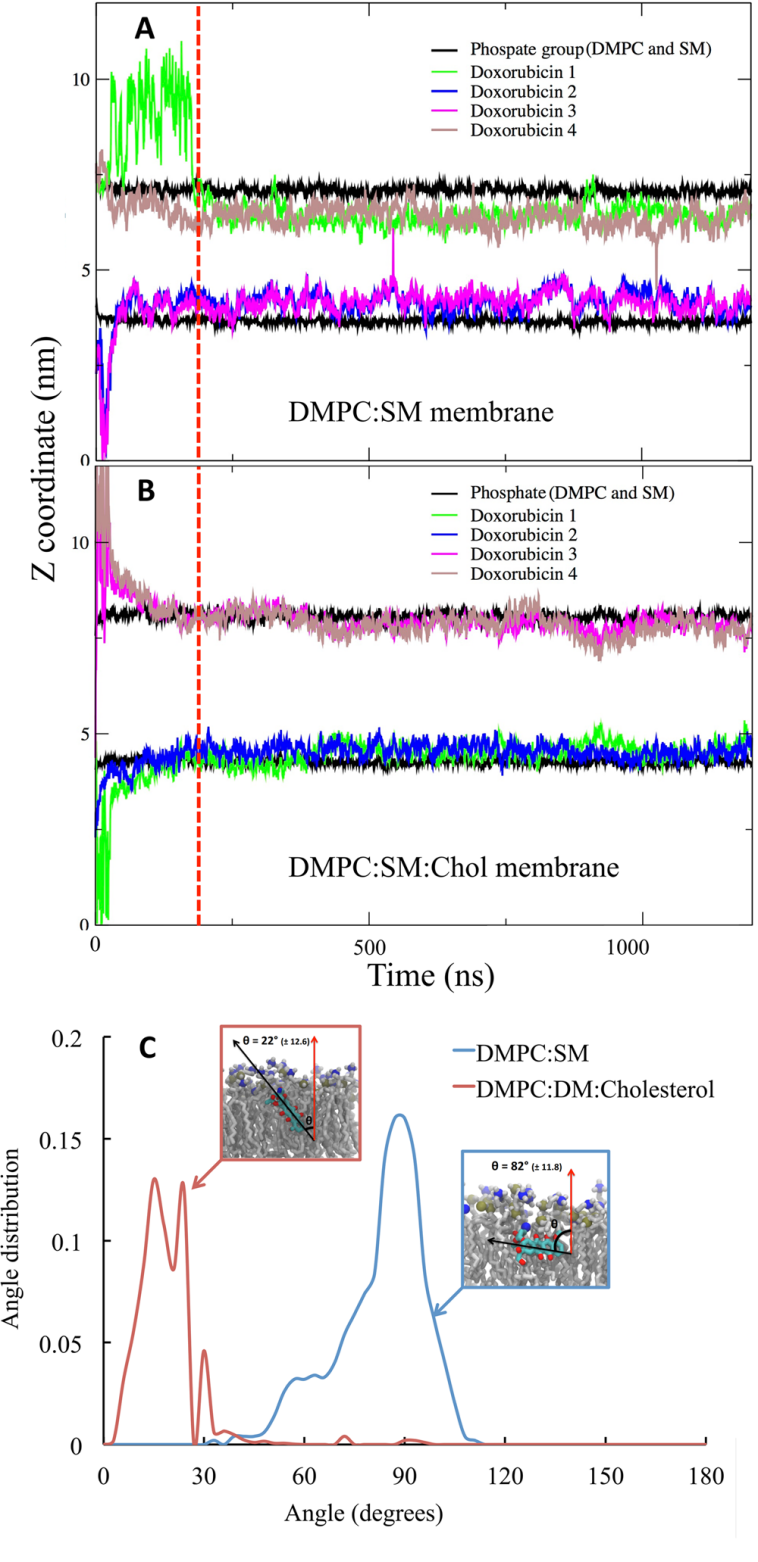
Partitioning of doxorubicin into the membrane bilayer models. The plot shows z-coordinate vs time for the center of mass of the phosphate group (of DMPC and SM) and doxorubicin molecules for (**A**) DMPC:SM [8:2] and (**B**) DMPC:SM:Chol [7:1.5:1.5] membranes. The red dotted line represents time point where systems were considered to be equilibrated, and for all analysis trajectory past this point (200 ns) was used. (**C**) The Angle distribution of doxorubicin in with membrane normal, in DMPC:SM (blue) and DMPC:SM:Chol (red) membranes. The error values represented are calculated as block errors.

We used MD simulation with all atom resolution to study the partitioning of doxorubicin into the DMPC:SM and DMPC:SM:Chol membrane bilayers. In all simulations, doxorubicin molecules were placed in the solvent at least 1.5 nm away from the lipid headgroups. The z-coordinate of the center of mass of doxorubicin molecule and z-coordinate of the center of mass of phosphate group vs time is shown in Fig. 6A and B. This analysis shows that the doxorubicin molecule partitions closer to the lipid-water interface in the presence of cholesterol, in good agreement with the derivative spectrophotometry studies. Furthermore, the angle of distribution of the doxorubicin molecule with the membrane normal (shown in red in Fig. 6C) shows that the orientation of the anticancer drug in the presence of cholesterol is significantly altered. As seen in Figs 6C and S2A, in the DMPC:SM membrane bilayer, the doxorubicin cyclic group (dihydroanthraquinone residue) is perpendicular to the membrane normal; however for the DMPC:SM:Chol membrane, the presence of cholesterol increases the degree of membrane ordering, resulting in an orientation change such that the average angle of doxorubicin to the membrane normal becomes 22 degrees. Such a difference in the doxorubicin orientation, concerning the two model membranes, might help to explain why alterations in membrane fluidity are more pronounced in the DMPC:SM bilayer. Furthermore, the increase in membrane ordering that results from the presence of cholesterol is traduced by an increase in membrane thickness, as shown in the Figure S2B. Also, the addition of cholesterol decreases the number of non-bonded contacts of lipids and cholesterol with doxorubicin as shown in Figure S3. However, in the presence of cholesterol, the number of hydrogen bonds between doxorubicin and both the lipids and cholesterol increases, as presented in Figure S4A and B. Also in Figure S4, we display the specific groups of doxorubicin involved in hydrogen bonding with lipids and both cholesterol (A) and water (B).

The high partitioning of doxorubicin with the two models can be explained by its ability to interact with the lipid tails as demonstrated in Figure S3 and to form hydrogen bonds with lipid headgroups (Figure S4).

In the previous simulation studies by Yacoub *et al*.^26^ it was demonstrated that the doxorubicin molecule has a significant effect on lipid ordering in the DPPC membrane bilayer. In this light, to investigate the effect of the insertion of a single doxorubicin molecule into the model membranes chosen for this study, we calculated the deuterium order parameter for the *sn*-2 chain of the DMPC molecule in both presence and absence of the doxorubicin molecule. The deuterium order parameter, S_CD_^27^, is a property of the bilayer that provides information concerning the level of lipid chain ordering. It can be obtained accurately from NMR experiments and is defined as follows:

1 $${{\rm{S}}}_{{\rm{CD}}}=(3/2\,{\cos }^{2}{{\rm{\theta }}}_{{\rm{i}}}\,-\,1/2)$$

where θ_i_ is the angle between the C–D bond (C–H in simulations) of the *i* carbon atom and the bilayer normal. The angular brackets denote averaging over time and over relevant C–D bonds in the bilayer.

**Figure 7:**
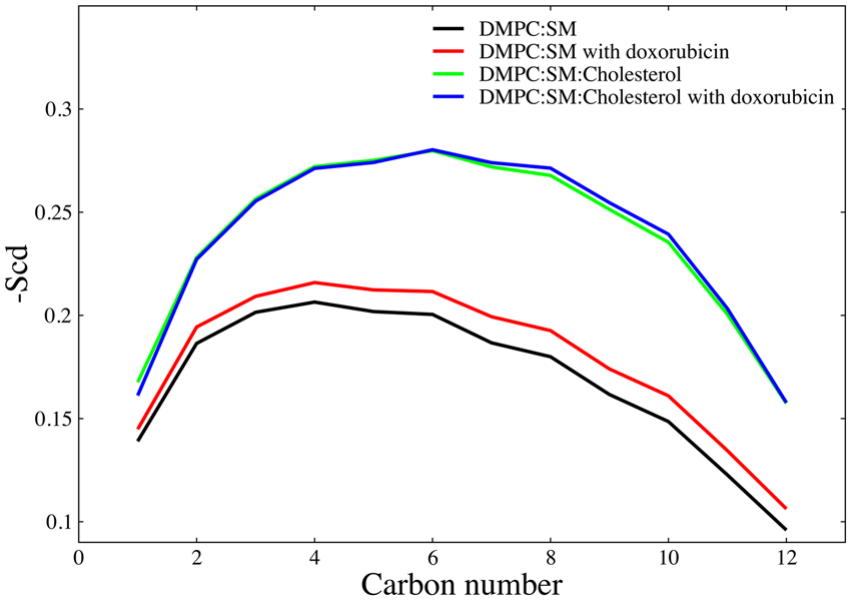
Order parameter of the *sn*-2 chain of DMPC molecule. Order parameter shows that the increase in lipid order parameter in DMPC:SM membrane is more pronounced as compared to DMPC:SM:Chol membrane in presence of doxorubicin molecule.

As shown in Fig. 7, we see that the change in the deuterium order parameter and thus, the degree of lipid chain ordering, that results from the presence of the doxorubicin molecule, is more pronounced for the DMPC:SM membrane than for the DMPC:SM:Chol membrane. This observation is in agreement with the fluidity measurements obtained from fluorescence anisotropy, as shown in Fig. 5.

**Figure 8:**
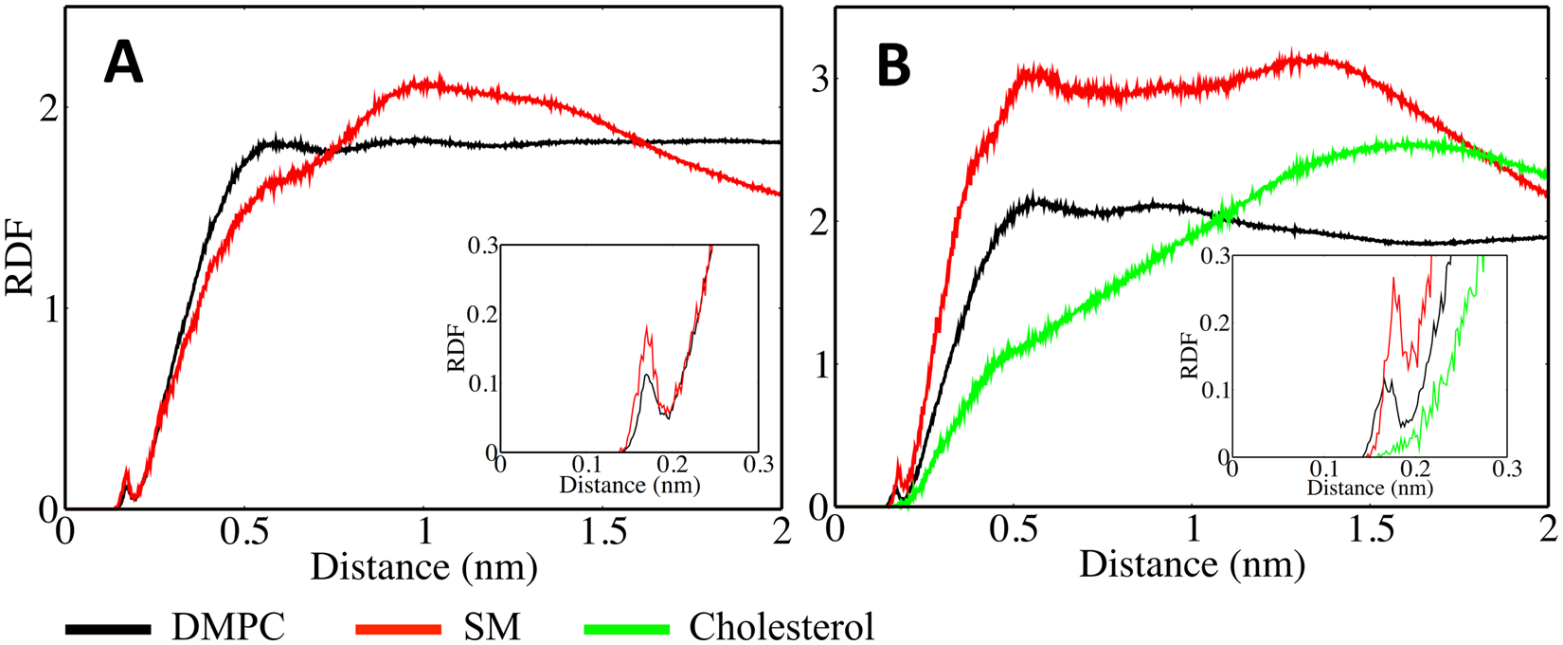
Molecular interactions of doxorubicin with individual components of the membrane bilayers: Radial distribution function of the DMPC, SM headgroups and cholesterol OH group from doxorubicin. (**A**) DMPC:SM [8:2] with doxorubicin and (**B**) DMPC:SM:Chol [7:1.5:1.5] with doxorubicin.

We thus observed that, as a result of the presence of cholesterol: 1) the orientation of the doxorubicin molecule in the membrane bilayer is altered, 2) the number hydrogen bonds between Doxorubicin and the membrane lipids is increased and 3) the extent of non-bonded interactions is decreased (Figure S3). In spite of this marked effect, the extent of direct interaction between cholesterol and doxorubicin was found to be relatively insignificant, as seen in Fig. 8. This might be explained by the fact that the OH group of cholesterol is masked by the polar head of the sphingomyelin in a typical “umbrella” effect^28^ and, thus, not available to interact with the doxorubicin molecule. In Fig. 8 the distribution of the measured distances between doxorubicin and DMPC, SM and Chol is shown. From this plot, it is clear that doxorubicin preferentially locates in close proximity to both the SM and DMPC molecules in the membrane. Hence the effect of cholesterol on the behavior of the membrane is significant even though this effect is not the result of direct interaction, but rather the change in the membrane structure that results from the presence of cholesterol.

## Conclusion

The present work investigated the molecular interactions and the behavior of an extensively used drug, doxorubicin, with model membranes composed of DMPC and SM, both in the presence (ordered) and absence (liquid-disordered) of Chol using combined *in vitro* and *in silico* models. Our goal was to shed light on the difference in behavior of doxorubicin in cholesterol rich ordered rafts from the rest of the biomembrane. The interaction of doxorubicin with a lipid membrane, i.e. how it positions in the membrane, was found to be strongly affected by the ordering effect of cholesterol. In all cases doxorubicin located within our model membranes, driven by non-bonded interactions (electrostatic, hydrophobic and Van der Waals interactions), as observed by both *in vitro* and *in silico* results. Furthermore, in the presence of cholesterol, the doxorubicin 1) preferentially partitions within the bilayer closer to the lipid-water interface and 2) displays an increased number of hydrogen bonds with both lipid and water molecules. Cholesterol is present in plasma membranes and modulates its physical properties. Any change produced as a result of drug-cholesterol interactions in modulating the function of cholesterol is of particular importance; this has been studied extensively in recent years^29^. In our studies, however, cholesterol was found to not interact directly with the drug and the effect of the presence of cholesterol was solely due to its the ordering effect in membrane structure. Collectively the *in vitro* and *in silico* results combine to build this picture, where it was possible to demonstrate that the presence of cholesterol does not reduce doxorubicin membrane partitioning, but it influences the drug’s ability to alter membrane fluidity. Furthermore, computational results show that cholesterol alters the behavior of the doxorubicin in the membrane without itself interacting with the doxorubicin: the effect is mediated through how it alters the membrane structure. This result can shed light on the doxorubicin chemoresistance problem as follows: 1) P-gp, the protein responsible for removal of xenobiotics like doxorubicin, preferential locates to lipid rafts and 2) our results now indicate that doxorubicin also preferentially locates to lipid rafts rich in cholesterol^11^, thus increasing their bioavailability to the efflux pump probably responsible for the reduced drug concentration inside the cell.

## Materials and Methods

In this work, we have studied synthetic models (liposomes) composed of 1,2-dimyristoyl-*sn*-glycero-3-phosphocholine (DMPC), sphingomyelin (SM) and cholesterol (Chol) were used in order to mimic specific membrane characteristics such as the presence of lipid rafts. Phosphatidylcholines are among the most abundant phospholipids of natural plasma membranes^4^ and were, for that reason, included in the mimetic models as the dominant constituent (DMPC). Sphingomyelin and cholesterol are important components of the cell membrane that undergo tight packing with phosphatidylcholines, forming the so called dynamic lipid rafts/nano-domains. These structures also control membrane fluidity and permeability, crucial for the preservation of its organization and, consequently, contributing to the cell membrane’s physiology and function^30^. Thereby, the ability of doxorubicin to partitioning into lipid bilayers (assessed by calculating the coefficient partition, Kp, using derivative spectrophotometry), its location within the membrane (by fluorescence quenching) and its effect in the membrane fluidity (through fluorescence anisotropy) were investigated. Molecular simulations were also performed, using the same lipid composition, in order to gain further insight regarding the interactions of doxorubicin with the studied model membranes.

### Reagents

Doxorubicin (DOX) was obtained from LC Laboratories (Woburn, Massachusetts, USA). The lipids 1,2-dimyristoyl-*sn*-glycero-3-phosphocholine (DMPC), sphingomyelin (SM) and cholesterol (Chol) were supplied by Avanti Polar Lipids, Inc. (Alabama, USA). The probes 1,6-diphenyl-1,3,5-hexatriene (DPH) and 1-(4-trimethylammoniophenyl)-6-phenyl-1,3,5-hexatriene (TMA-DPH) were purchased from Molecular Probes (Invitrogen Corporation, Carlsbad, California, USA). All reagents were used without further purification. Drug solutions were prepared with Hepes buffer (10 mM, pH 7.4) and the ionic strength was adjusted with NaCl (I = 0.1 M) to mimic physiological conditions. The buffer was prepared using double-deionized water (conductivity less than 0.1 μS cm^−1^).

### Liposomes Preparation

Liposomes were prepared according to the adapted classic thin film hydration method^31^. Briefly, the lipids DMPC:SM [8:2] and DMPC:SM:Chol [7:1.5:1.5] were dissolved in chloroform/methanol (3:2 v/v) and the organic solvents were evaporated under a stream of nitrogen in a rotative evaporator (Buchi R-200, equipped with a thermostated bath Buchi R-490, Switzerland). The obtained dried lipid film was dispersed in Hepes (pH 7.4) and the mixture was vortexed to yield multilamellar vesicles (MLVs). Large unilamellar vesicles (LUVs) were obtained by extruding the MLVs eleven times (extruder supplied by Lipex Biomembranes Inc., Vancouver, Canada) using policarbonate filters with a diameter pore of 100 nm (Nucleopore) at 60 °C (temperature above the main phase transition temperature of the lipids). Labeled liposomes were identically prepared, except that the fluorescence probe (DPH or TMA-DPH) was co-dissolved with lipids in the organic solvents mixture to give a probe/lipid molar ratio of 1:300 (prevent changes in the membrane’s structure).

### Determination of Partition Coefficients by Derivative Spectrophotometry

Partition coefficient (Kp) of doxorubicin between liposomes/buffer systems was assessed by derivative spectrophotometry, as described in previous works^21, 31, 32^. For these studies, a set of samples containing a fixed concentration of doxorubicin (40 μM) and increasing concentrations of lipids (in the range of 0–1250 μM) were prepared. The equivalent references were identically prepared but without the drug. All suspensions were incubated during 30 min at 37 °C. The absorption spectra of all solutions were recorded at 37 °C using a multidetection microplate reader (Synergy™ HT, BioTek Instruments Inc, Winooski, VT, USA). The Kp values were calculated from the third derivative spectra using a developed routine, the *Kp Calculator* ^33^, by fitting equation 1 to the experimental data (D_T_ vs [L]) using a nonlinear regression method:

2 $${D}_{{\rm{T}}}\,={D}_{{\rm{W}}}+\,\frac{({D}_{{\rm{m}}}-{D}_{{\rm{w}}}){K}_{{\rm{p}}}[L]{V}_{{\rm{m}}}}{1+{K}_{{\rm{p}}}[L]{V}_{{\rm{m}}}}$$

where, D_T_ is the third derivative intensity (D = d^n^Abs/dλ^n^) obtained from the absorbance values of the total amount of doxorubicin (D_T_), D_m_ and D_w_, the doxorubicin distributed in the lipid membrane phase and in the aqueous phase respectively. [L] represents the lipid concentration (in M) and V_m_ is the lipid molar volume, calculated from the weighted molecular mean and the reported volume of lipids, for the biomimetic membranes (DMPC:SM and DMPC:SM:Chol) were 0.682 and 0.633 L.mol^−1^, respectively. The Kp values obtained are dimensionless and presented as the mean ± standard deviation calculated from at least three independent assays.

### Drug Location Studies Evaluated by Fluorescence Quenching

Doxorubicin location within the model systems was assessed by fluorescence studies that included steady-state and time-resolved measurements, according to an already described protocol^34^. These experiments were conducted by incubation of the anticancer-drug with DPH and TMA-DPH labeled liposomes. The samples containing a fixed concentration of lipid (500 μM) and increasing concentrations of doxorubicin (0, 40 and 75 μM), were incubated at 37 °C during 30 min in the dark. The fluorescence measurements were carried out at 37 °C, at the excitation and emission wavelengths defined as 357 nm and 429 nm, and 359 nm and 429 nm, for DPH and TMA-DPH, respectively. Fluorescence steady-state measurements were performed in a Jasco FP-6500 spectrofluorometer (Jasco, Great Dunmow, UK) equipped with a constant temperature cell holder and all data were recorded in a 1 cm path length cuvette. For each measurement, the fluorescence emission was automatically acquired during 30 s. The fluorescence intensity values obtained were corrected for inner filter effects (quencher absorbance) at the excitation wavelength^35^. The fluorescence time-resolved measurements were performed using a Fluorolog Tau-3 Lifetime system. The modulation frequencies were acquired between 10 and 200 MHz, with an integration time of 10 s. The fluorescence emission was detected with a 90° scattering geometry. All measurements were made using Ludox as a reference standard (τ = 0.00 ns).

### Membrane Fluidity Studies Evaluated by Fluorescence Anisotropy

The doxorubicin’s effect on the membrane microviscosity, in the different model membranes, was assessed by fluorescence anisotropy studies. Samples were prepared with a fixed concentration of lipid (500 μM), labeled with DPH or TMA-DPH, and increasing concentrations of doxorubicin (0, 40 and 75 μM), and incubated during 30 min at 37 °C in the dark. The steady-state fluorescence anisotropy values (r_s_) were recorded between 10 and 60 °C with intervals of 2 °C. The experiments were performed in a Jasco FP-6500 spectrofluorometer with polarizers inserted (excitation/emission wavelengths were set as described for each fluorescent probe). The results obtained are presented as the mean ± standard deviation calculated from at least three independent assays.

### Molecular Dynamics (MD) simulations

All-atom molecular dynamics simulations of doxorubicin molecule were performed with 2 membrane bilayers, with the following lipid compositions, DMPC:SM [8:2] DMPC:SM:Chol [7:1.5:1.5]. For lipid molecules S-lipids forcefield parameters were used^36–38^. For water molecules we used the TIP3P model compatible with the S-lipids forcefield set. Periodic boundary conditions with minimum image convention were used in all directions. Covalent bond lengths were preserved through the linear constraint solver (LINCS) algorithm^39^. For all systems the temperature was controlled using the Nosé-Hoover thermostat with solvent and solute controlled independently^40, 41^. Pressure was controlled using the Parrinello-Rahmann barostat with semi-isotropic control^42^. Lennard-Jones interactions were cut off at 1.4 nm and for the electrostatic interactions the particle mesh Ewald method (PME)^43^ was used. For all simulations physiological pressure and temperature were used (1 Bar and 37 °C). The GROMACS open source software package, version 4.6.5, was used^44^ for all Molecular Dynamics (MD) simulations, along with all quantitative analysis. Visualization of the trajectories was performed using the Visual Molecular Dynamics (VMD) package^45^. For doxorubicin, the partial charges were derived with RESP^46^ procedure implemented in ANTECHAMBER^47^. All the simulation systems were simulated for 1.2 microsecond. The first 200 ns were considered as an equilibration phase and, hence all the analysis was performed on the latter 1 microsecond trajectories.

### Statistical analysis

Statistical analysis was performed using IBM® SPSS® Statistics software (v.20.0.0.0, IBM, Armonk, NY, USA). The measurements were repeated at least three times and data was expressed as mean ± standard deviation (SD). Data was statistically analyzed through the one-way analysis of variance (ANOVA) method and differences between groups were compared by Bonferroni and Tukey post-hoc tests in which a p value lower than 0.05 (p < 0.05) was considered statistically significant.

## Electronic supplementary material

Supporting information
