# Supplementary material for: Influence of doxorubicin on model cell membrane properties: insights from in vitro and in silico studies
Source: Sci Rep. 2017 Jul 24;7:6343. doi: 10.1038/s41598-017-06445-z (PMC5524714; doi:10.1038/s41598-017-06445-z)
Supplement: Supplementary file 1 — Supporting information [file 41598_2017_6445_MOESM1_ESM.pdf]

Supporting Information

Influence of doxorubicin on model cell membrane properties: insights from *in vitro* and *in silico* studies

Ana Catarina Alves[1+], Aniket Magarkar [2,3+], Miguel Horta[1], José L.F.C. Lima[1], Alex Bunker[3], Cláudia Nunes[1*], and Salette Reis[1]

[1] UCIBIO, REQUIMTE, Departamento de Ciências Químicas, Faculdade de Farmácia, Universidade do Porto, Portugal

[2] Institute of Organic Chemistry and Biochemistry, Academy of Sciences of the Czech Republic, Flemingovo nám. 2, 16610 Prague 6, Czech Republic

[3] Faculty of Pharmacy, University of Helsinki, Viikinkaari 5E, Helsinki, 00014 Finland

[+]authors contributed equally

*Corresponding author:

REQUIMTE, Departamento de Ciências Químicas, Faculdade de Farmácia, Universidade do Porto

Rua de Jorge Viterbo Ferreira n.º 228

4050-313 Porto, Portugal

cdnunes@ff.up.pt

Tel: +351-220428672

Fluorophore quenching – Theoretical background

The drug efficiency to quench the fluorophore is related to its proximity to the probe. In this sense, because the fluorophore is inserted in the membrane, only the drug that partitions into the lipid phase will be able to act as a quencher. In order to determine the doxorubicin quenching efficiency, it is important to calculate its membrane concentration ($[\mathrm{Q}]_\mathrm{m}$) that is given by the equation 1:

$$[\mathrm{Q}]_\mathrm{m} = \frac{K_\mathrm{p}[\mathrm{Q}]_\mathrm{T}}{K_\mathrm{p}\alpha_\mathrm{m} + (1 - \alpha_\mathrm{m})} \qquad (1)$$

where α_m is the volume fraction of membrane phase ($\alpha_\mathrm{m} = V_\mathrm{m}/V_\mathrm{T}$; V_m and V_T represent the volumes of the membrane and water phase, respectively) and Q_T represents the total drug concentration used.

The ability of doxorubicin to quench DPH and TMA-DPH probes was analyzed according to the modified Stern-Volmer equation (2):

$$\frac{I_0}{I} = K_\mathrm{SV}\,[Q]_\mathrm{m} + 1 = \frac{\tau_0}{\tau} \qquad (2)$$

where I and I_0 are the steady state fluorescence intensities with and without the quencher (doxorubicin), respectively; K_SV is the Stern-Volmer constant; ($[\mathrm{Q}]_\mathrm{m}$) is the doxorubicin membrane concentration; and τ_0 and τ are the fluorophore lifetime in the absence and presence of doxorubicin, respectively. The molecular contact between the probe (fluorophore) and the drug (quencher) can be due to different molecular interactions and can be observed by the decrease in the fluorescence intensity (fluorescence quenching). Furthermore, the quenching process can result from collisional encounters between the fluorophore and quencher, and is therefore called collisional or dynamic quenching, or can be due to complex formation, which is called static quenching.[31] In order to understand which type of quenching mechanism occurs, the dynamic quenching constant (K_D) was calculated from the slope of the Stern-Volmer plots obtained by lifetime fluorescence measurements (τ_o/τ) versus the doxorubicin membrane concentration ($[\mathrm{Q}]_\mathrm{m}$) (equation 3):[31]

$$\frac{\tau_0}{\tau} = K_\mathrm{D}[Q]_\mathrm{m} + 1 \qquad (3)$$

The static quenching constant (K_S) was also determined from the following relation between the K_SV and K_D constants (equation 4):[31]

$$K_\mathrm{SV} = K_\mathrm{S} + K_\mathrm{D} \qquad (4)$$

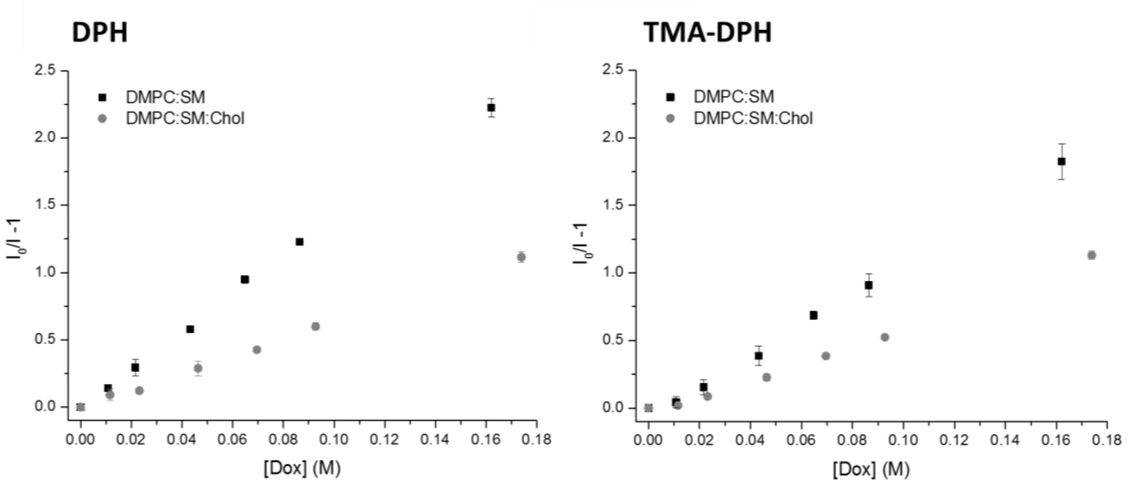

Figure S1: Stern–Volmer plots of the probes DPH and TMA-DPH obtained by steady-state fluorescence measurements (I_0/I-1) for DMPC:SM (■) and DMPC:SM:Chol (●) model membranes with increasing doxorubicin concentrations, at pH 7.4 and 37 °C.

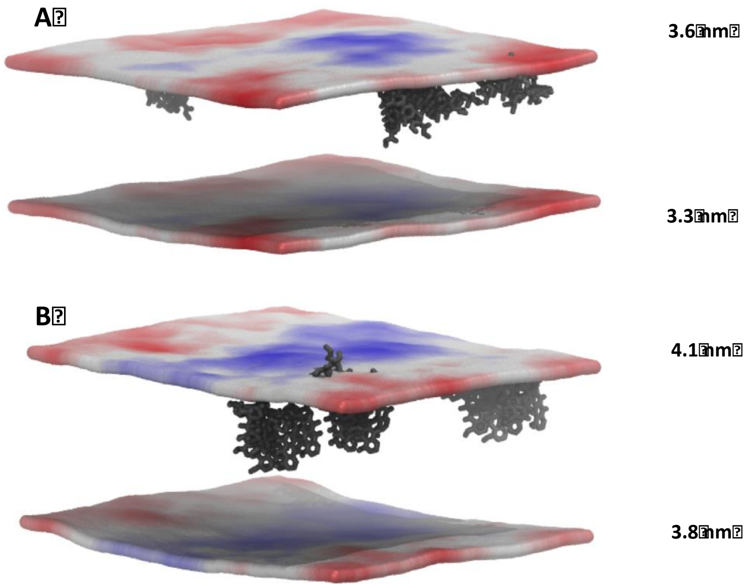

Figure S2: Membrane thickness of (A) DMPC:SM and (B) DMPC:SM:Chol and average positioning of doxorubicin conformation sampled over 1 microsecond of simulation trajectory.

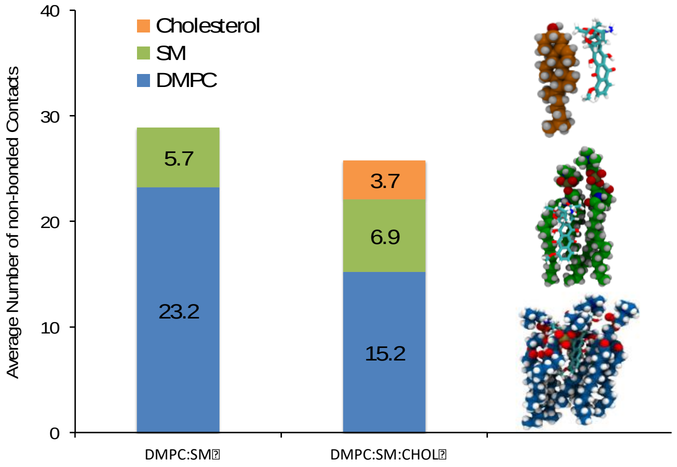

Figure S3: Number of non-bonded contacts of doxorubicin with DMPC, SM and Chol. These atoms are involved in electrostatic and hydrophobic interactions with the doxorubicin molecule.

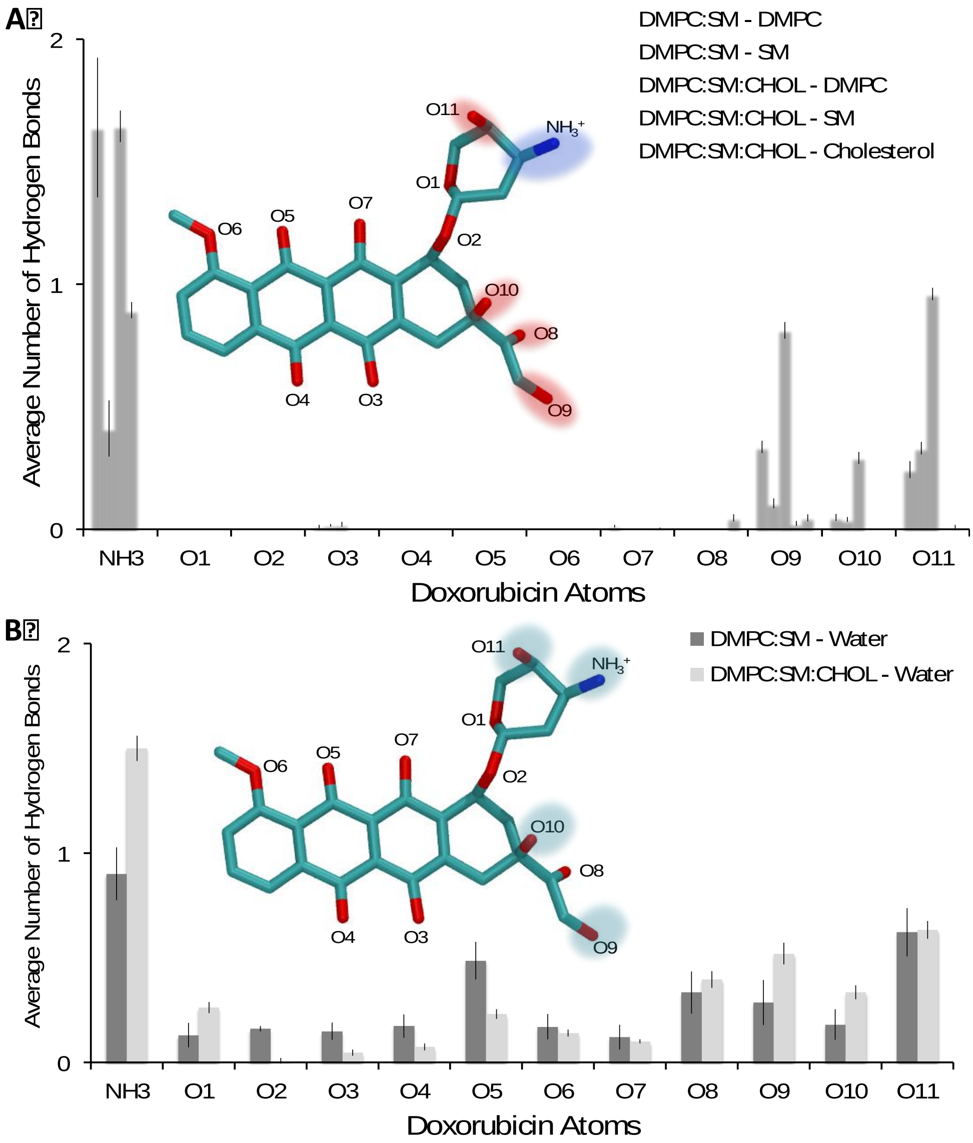

Figure S4: Average number of hydrogen bonds of doxorubicin in DMPC:SM and DMPC:SM:CHOL membranes with (A) lipids and cholesterol and (B) with water. The number of hydrogen bonds were normalized by number of doxorubicin molecules.
